# Targeting Macrophages as a Therapeutic Option in Coronavirus Disease 2019

**DOI:** 10.3389/fphar.2020.577571

**Published:** 2020-10-29

**Authors:** Maria Gracia-Hernandez, Eduardo M. Sotomayor, Alejandro Villagra

**Affiliations:** ^1^ Department of Biochemistry and Molecular Medicine, School of Medicine and Health Sciences, The George Washington University, Washington, DC, United States; ^2^ The George Washington University Cancer Center, School of Medicine and Health Sciences, The George Washington University, Washington, DC, United States

**Keywords:** macrophage, COVID-19, coronavirus, fibrosis, cytokine storm

## Abstract

Immune cells of the monocyte/macrophage lineage are characterized by their diversity, plasticity, and variety of functions. Among them, macrophages play a central role in antiviral responses, tissue repair, and fibrosis. Macrophages can be reprogrammed by environmental cues, thus changing their phenotype during an antiviral immune response as the viral infection progresses. While M1-like macrophages are essential for the initial inflammatory responses, M2-like macrophages are critical for tissue repair after pathogen clearance. Numerous reports have evaluated the detrimental effects that coronaviruses, e.g., HCoV-229E, SARS-CoV, MERS-CoV, and SARS-CoV-2, have on the antiviral immune response and macrophage functions. In this review, we have addressed the breadth of macrophage phenotypes during the antiviral response and provided an overview of macrophage-coronavirus interactions. We also discussed therapeutic approaches to target macrophage-induced complications, currently under evaluation in clinical trials for coronavirus disease 2019 patients. Additionally, we have proposed alternative approaches that target macrophage recruitment, interferon signaling, cytokine storm, pulmonary fibrosis, and hypercoagulability.

## Introduction

Macrophages play multiple roles in the innate immune system. They can phagocytose bacteria and viruses to trigger an immune response, as well as promote tissue homeostasis and regeneration (Gordon and Taylor, 2005). Macrophages can be found in many tissues and organs, e.g., lungs, liver, spleen, and lymph nodes, among others. Tissue-resident macrophages can coexist with monocyte-derived macrophages that are recruited to inflamed tissues (Gordon and Taylor, 2005). Cytokines present in the tissue microenvironment cause the phenotypic programming of these monocyte-derived macrophages, which can be classified into two subtypes: classically activated (M1-like), or alternatively activated (M2-like) (Gordon and Taylor, 2005; Sica and Mantovani, 2012; Wang et al., 2014). These M1-M2 phenotypes differ in their cytokine secretion profiles and functions. Briefly, M1-like macrophages are associated with microbicidal activity, pro-inflammatory cytokine production, and immune response, while M2-like macrophages are important for angiogenesis, tissue maintenance, repair, and secretion of anti-inflammatory cytokines. Both phenotypes are important during viral infections, as M1-like macrophages are needed for pathogen detection to initiate the initial inflammatory response, and M2-like macrophages are necessary to heal the damaged tissue at the aftermath of the viral infection. Therefore, macrophage recruitment, polarization, and functions are critical for a balanced and controlled antiviral immune response that leads to successful pathogen clearance without tissue damage.

Macrophages can recognize viruses in different families, such as Orthomyxoviridae (Kato et al., 2006; Loo et al., 2008), Picornaviridae (Gitlin et al., 2006; Kato et al., 2006), and Coronaviridae (Roth-Cross et al., 2008)*.* The Coronaviridae family of viruses belongs to the *Nidovirales* order, which consists of enveloped, positive-sense RNA viruses that contain some of the largest RNA genomes ever identified (Fehr and Perlman, 2015). Coronaviruses (CoVs) are characterized by the presence of spike proteins that project from the surface of the virion, thus giving them the appearance of a solar corona. CoVs are comprised of four main structural proteins, which include the spike, membrane, envelope, and nucleocapsid proteins (S, M, E, and N, respectively). The S protein is used for the attachment of the virus to the target cell in the host, while the M, E, and N proteins have additional structural functions. CoVs also encode for multiple nonstructural (NS) proteins that may help promote infection, replication, and viral assembly (Fehr and Perlman, 2015).

Prior to the SARS-CoV-2 outbreak at the end of 2019 in Wuhan, China, and the SARS-CoV outbreak in China in 2002, CoVs were only associated with mild respiratory infections (Fehr and Perlman, 2015). HCoV-229E, HCoV-NL63, HCoV-OC43, and HCoV-HKU1 can cause upper respiratory tract infections similar to the common cold, while SARS-CoV, MERS-CoV, and SARS-CoV-2 are responsible for more severe cases of pneumonia and life-threatening conditions (Li et al., 2020). To illustrate, the severe acute respiratory syndrome (SARS) outbreak in 2002–2003 reported about 8,100 cases and less than 800 deaths (Graham et al., 2013; Fehr and Perlman, 2015). The Middle East respiratory syndrome (MERS) outbreak occurred in Saudi Arabia in 2012, where it affected around 1,200 people, with a 40% mortality rate (Fehr and Perlman, 2015; Zumla et al., 2015; Li et al., 2020). However, SARS-CoV-2, the causative agent of coronavirus disease 2019 (COVID-19), is the most widely spread coronavirus-related infection known to date, with more than 25 million cases and more than 800,000 deaths as of August 31, 2020, according to the WHO. Although there is emerging data on the origin of SARS-CoV-2, comparative genomic data analysis suggests potential ways by which it could have originated (Andersen et al., 2020). SARS-CoV-2 is 82% similar to human SARS-CoV, 89% similar to the bat SARS-like-CoVZXC21 (Chan et al., 2020), and 96.2% similar to the bat coronavirus RaTG13 (Zhou et al., 2020). Despite these similarities, COVID-19 and SARS have important differences, such as transmissibility, severity, and infectious period (Wilder-Smith et al., 2020), which need to be considered when looking for therapeutic options for COVID-19 patients, as there are no vaccines available at the time of this review.

SARS-CoV-2 infects cells by attaching to angiotensin-converting enzyme 2 (ACE2), like other CoVs such as SARS-CoV, and HCoV-NL63 (Li et al., 2003; Kuba et al., 2005; Fehr and Perlman, 2015, 63; Hoffmann et al., 2020). ACE2 is expressed in multiple cell populations that can be found in the lungs, including alveolar type II pneumocytes and macrophages (Xu H. et al., 2020; Zhang D. et al., 2020), suggesting that these CoVs can potentially infect ACE2+ macrophages and negatively affect the antiviral immune response they can elicit. Although multiple reports on SARS-CoV-2 and macrophage interactions that are discussed in this review have not been peer-reviewed yet, they suggest that CoVs can negatively affect and diminish macrophage function during the antiviral response. This may lead to the development of the life-threatening conditions that are observed in severely ill COVID-19 patients like cytokine storm and pulmonary fibrosis (Jose and Manuel, 2020; Yuen et al., 2020; Mehta P. et al., 2020; Xu Y.-H. et al., 2020), as these processes are tightly regulated by macrophages.

Hence, modulating macrophage function and recruitment during the CoV infection process is a potential therapeutic approach to improve the antiviral immune response and decrease the life-threatening conditions observed in severe cases. In this review, we evaluate the role of macrophage phenotypes at different stages of the viral infection and describe macrophage-coronavirus interactions. In addition, we summarize ongoing clinical trials for COVID-19 patients that target macrophage function, as well as propose alternative approaches.

## The Role of Macrophage Phenotypes During Viral Infections

Diversity and plasticity are characteristics of cells of the monocyte/macrophage lineage (Guiducci et al., 2005; Biswas and Mantovani, 2010; Sica and Mantovani, 2012; Wang et al., 2014). Macrophages can be classified into M1-like or M2-like, although this is a simplistic classification as their phenotype can switch between these subtypes and even adopt hybrid characteristics (Varol et al., 2015; Shapouri-Moghaddam et al., 2018). The tissue microenvironment reprograms macrophages after their infiltration. For example, anti-tumor M1 macrophages are reprogrammed toward the pro-tumoral M2 phenotype after transplantation due to the dominant anti-inflammatory niche promoted by tumor cells in the tumor microenvironment (Andreesen et al., 1998; Mills et al., 2016). Similarly, M1-like macrophages are more prominent in the initial stages of the antiviral immune response, adopting an M2-like phenotype at the later stages to initiate the resolution of inflammation.

**FIGURE 1:**
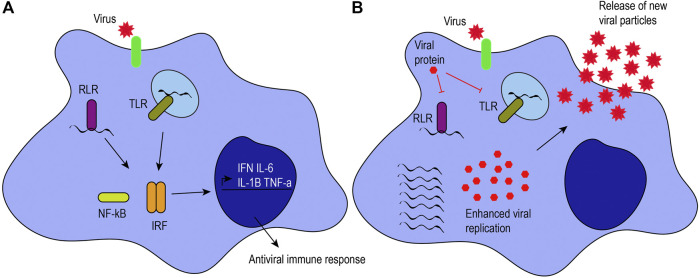
Antiviral response induced by macrophages. **(A)** Viruses can infect target cells by interacting with their receptors (green) or by phagocytosis. Macrophages can recognize viral proteins and genomes to trigger an antiviral immune response. When RIG-I-like receptors (RLRs) and Toll-like receptors (TLRs) recognize viral genomes, they activate the NF-κB and interferon regulatory factor signaling pathways, leading to the production of proinflammatory cytokines (i.e., IL-6, IL-1β, and TNF-α) and interferons (IFN). **(B)** Viruses have mechanisms to hijack the antiviral immune response. For example, some viral proteins can inhibit TLRs and RLRs, inhibiting the activation of those signaling pathways and enhancing viral pathogenesis, as illustrated by an increase in viral genome replication, viral protein expression, and the release of new viral particles.

M1-like macrophages are associated with microbicidal activity, pro-inflammatory cytokine production, and immune response. They have increased production of inflammatory mediators, reactive oxygen species, costimulatory molecules, antigen presentation, and phagocytic activity. They are characterized by the expression of major histocompatibility complex class II molecules, CD80, IDO (indoleamine-pyrrole 2,3-dioxygenase), and NOS2 (nitric oxide synthase 2), among others (Martinez and Gordon, 2014). Additionally, recognition of pathogen-associated molecular patterns by pattern recognition receptors confers an M1-like phenotype, as pro-inflammatory cytokines and interferons are secreted (Figure 1A).

Pattern recognition receptors expressed on macrophages and other innate immune cells include Toll-like receptors (TLRs 2, 3, 4, 7/8, and 9) as well as retinoic acid-inducible gene-I-like receptors (RLRs), among others (Lester and Li, 2014). TLRs can be present on the plasma membrane or endosomes of macrophages, depending on their ligand specificity for viral proteins or genomes. For example, TLR2 and TLR4 recognize both structural and NSP proteins, such as hemagglutinin or NSP4 (Kurt-Jones et al., 2000; Bieback et al., 2002; Mogensen and Paludan, 2005; Ge et al., 2013). TLR3 responds to double-stranded RNA (dsRNA), while TLR7/8 recognizes viral single-stranded RNA, and TLR9 recognizes unmethylated CpG islands in viral DNA. Similarly, RLRs such as RIG-I and MDA-5 can distinguish between host and viral genomes. RIG-I recognizes uncapped, 5’ triphosphorylated viral RNA, while MDA-5 senses long dsRNA (Kato et al., 2006; Loo et al., 2008). RIG-I also recognizes short dsRNA that can be formed during viral replication (Weber et al., 2006; Targett-Adams et al., 2008). TLR, RIG-I or MDA-5 signaling activates interferon regulatory factors and NF-κB to promote the expression of pro-inflammatory cytokines and interferons (Ohman et al., 2009; Loo and Gale, 2011; Brisse and Ly, 2019). To illustrate, alveolar macrophages are the primary producers of interferon (IFN)-α through RLRs in pulmonary infections caused by RNA viruses (Kumagai et al., 2007). The secretion of inflammatory cytokines (e.g., IL-6, TNF-α, and IL-12) and interferons by M1-like macrophages stimulates T helper type 1 cell activity to promote cell-mediated immune responses against intracellular infections caused by viruses and bacteria (Romagnani, 2000). T helper type 1 cells release IFN-γ, TNF-β, and IL-2 that, in turn, activate macrophages and phagocyte-dependent responses (Romagnani, 2000).

After the inflammatory response is induced, M1-like macrophages switch to the M2-like phenotype to initiate the resolution of inflammation, maintain tissue integrity, and return to homeostasis. This phenotypic switch is characterized by decreased production of inflammatory mediators and increased release of anti-inflammatory cytokines such as IL-10 or transforming growth factor beta (TGF-β) (Fadok et al., 1998). M2-like macrophages secrete anti-inflammatory cytokines, and play essential roles in angiogenesis, tissue maintenance and repair, matrix remodeling, vascularization, immunosuppression, tumor progression, and immune tolerance (Sica and Mantovani, 2012; Wang et al., 2014). M2-like macrophages can be induced by IL-4, IL-13, IL-10, or TGF-β and are characterized by the expression of markers like CD163, CD206, CD23, YM1 (chitinase-like 3), FIZZ-1 (resistin-like molecule alpha-1), and ARG-1 (arginase-1) (Martinez and Gordon, 2014). M2-like macrophages recruit regulatory T cells to promote immunosuppression, and promote Th2 cell responses, leading to the secretion of IL-4, IL-5, IL-6, IL-9, IL-10, and IL-13 to trigger antibody responses, eosinophil accumulation, and inhibit phagocytic activity (Romagnani, 2000).

M2-like macrophages participate in the resolution of inflammation by playing direct and indirect roles in tissue healing and remodeling. They produce different components of the extracellular matrix (ECM), such as types I and III collagen and matrix metallopeptidases (MMPs) (Feng et al., 2019), or transition into collagen-producing fibroblasts through a macrophage-myofibroblast transition process regulated by TGF-β (Wang et al., 2016). M2-like macrophages can also secrete proangiogenic factors such as vascular endothelial growth factor or platelet-derived growth factor to promote vascularization of the healed tissues (Braga et al., 2015). Indirect roles include stimulating fibroblasts through TGF-β (Braga et al., 2015; Sheng et al., 2018) to promote ECM deposition, wound healing, and scar formation (Feng et al., 2019).

Despite their differences, M1-and M2-like macrophages share some characteristics. For example, M1-like are also associated with wound healing properties by secretion of MMPs and CCL2 (Braga et al., 2015; Feng et al., 2019). The latter can recruit fibrocytes that differentiate into fibroblasts to help with ECM deposition. However, M1-like macrophages’ wound healing properties can be “pathologic” due to the production of inflammatory mediators like interferons, IL-1β, or IL-6 (Feng et al., 2019). In addition, these inflammatory cytokines can also affect the M2-like phenotype (Sica and Mantovani, 2012). For instance, IL-6 can promote an M2-like phenotype (Braune et al., 2017; Sanmarco et al., 2017; Yin et al., 2018) and serve as a “brake” for macrophage activation as a mechanism to protect tissues from overactive immune responses (Luig et al., 2015). This suggests that cytokines secreted by M1-like macrophages promote phenotype reprogramming toward the M2-like.

**FIGURE 2:**
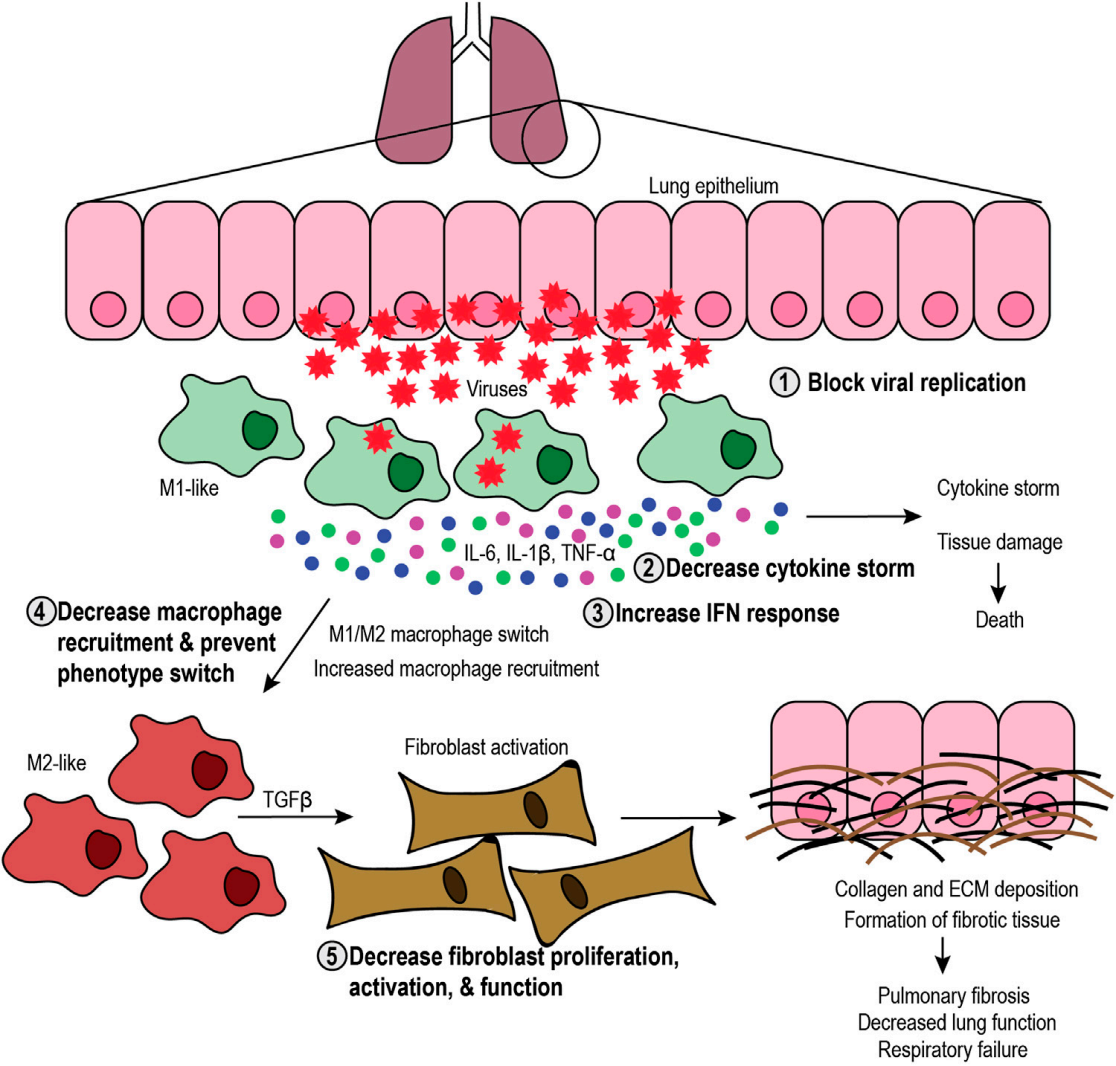
Targeting the SARS-CoV-2 infection process. Schematic representation of the infection process and the role of macrophages, including the steps that can be targeted. Viral infection in the lung epithelium causes cell lysis and recruitment of M1-like macrophages that elicit an antiviral immune response and secrete proinflammatory cytokines (interleukin-6 (IL-6), IL-1β, and tumor necrosis factor-α (TNF-α), leading to cytokine storm and subsequent tissue damage or death. Tissue damage further increases macrophage recruitment and can induce phenotype switch toward the M2-like phenotype. M2-like macrophages secrete transforming growth factor *β* (TGFβ), which activates fibroblasts. In turn, fibroblasts produce collagen and extracellular matrix (ECM), which leads to formation of fibrotic tissue, decreased lung function and respiratory failure. Therefore, therapeutic approaches include 1) blocking viral replication, 2) decreasing the cytokine storm, 3) increasing the interferon (IFN) response, 4) decreasing macrophage recruitment and phenotype switch, and 5) decreasing fibroblast proliferation, activation, and function.

M1-like macrophage function is critical at the early stages of the infection process to elicit the initial inflammatory response and stimulate immune cells in autocrine and paracrine fashions, while M2-like macrophages are necessary for the resolution of the inflammatory response and healing the damaged tissue. Disruption of this balance can lead to severe complications in patients, including cytokine storm, tissue damage, and fibrosis (Figure 2). If M1-like function is altered, excessive secretion of pro-inflammatory cytokines can lead to a “cytokine storm” that causes systemic inflammation and tissue damage. For instance, overproduction of IL-6 and other pro-inflammatory cytokines leads to a cytokine storm in patients, increasing the risk of multiorgan failure, vascular permeability, and consequent death (Meduri et al., 1995a; Meduri et al., 1995b; Jose and Manuel, 2020). Interestingly, sustained IL-6 production is linked to an increase in pathogenesis and viral persistence (Akira and Kishimoto, 1992; Khong et al., 2011; Hou et al., 2014). Besides IL-6, damage-associated molecular patterns released from infected host cells cause the phenotype reprogramming of M1-like macrophages toward the M2-like phenotype. In addition to inducing the secretion of pro-inflammatory cytokines by M1-like macrophages and other APCs (Tsai et al., 2014; Dapat et al., 2017), damage-associated molecular patternss also promote the secretion of IL-4 and IL-13, inducing the M2-like phenotype with wound healing, pro-angiogenic, and pro-fibrotic functions (Wynn et al., 2013).

Hence, imbalanced M1-like functions create a positive feedback loop of inflammation that leads to cytokine storm and tissue damage that, in turn, increases the activity of M2-like macrophages. M2-like function induces the proliferation and activation of fibroblasts that secrete collagen and other ECM components, thus exacerbating the formation of fibrotic tissue. Fibrosis has long-lasting, harmful effects on patients, potentially causing organ failure and death. Therefore, it is critical to understand the role of M1-like and M2-like macrophages during viral infections to develop therapies that aim to maintain the balance between their functions.

## Macrophage-Coronavirus Interactions

Viruses can hijack and downregulate the antiviral immune response elicited by macrophages to enhance viral replication and pathogenesis (Figure 1B). CoVs have developed different mechanisms to evade the host immune response, to downregulate the inflammatory response, and to increase their pathogenesis. For example, CoVs have NSPs that help them escape the immune response by evading dsRNA sensors (Deng and Baker, 2018) and downregulating type I and III interferons (Deng et al., 2019). In this section, we provide an overview of the effects of HCoV-229E, SARS-CoV, MERS-CoV, and SARS-CoV-2 upon macrophages.

One commonality between HCoV-229E, SARS-CoV, MERS-CoV, and SARS-CoV-2 is their ability to cause respiratory issues, which can be associated with high morbidity and mortality. These severe outcomes may be linked to the imbalance of macrophage populations in the lungs during CoV infections. Tissue-resident alveolar macrophages constitute the first line of defense in the lungs, thus playing essential roles in immune surveillance and maintaining tissue integrity (Hussell and Bell, 2014). Alveolar macrophages originate in the yolk sac, where they can coexist with recruited monocyte-derived macrophages (Wynn et al., 2013). Alveolar macrophages have the ability for self-renewal and persist over time. However, environmental challenges such as viruses, bacteria, cigarette/tobacco smoke, and contaminants can change the composition of macrophages in the lung, leading to a decrease in alveolar macrophages and an increase in monocyte-derived macrophages (Morales-Nebreda et al., 2015). Alveolar macrophages express proteins such as CD200, SIRPα (signal regulatory protein alpha), scavenger receptors, and TGF-β to facilitate the non-inflammatory clearance of pathogens, debris, and apoptotic cells without disrupting lung function (Morales-Nebreda et al., 2015). However, severe infections can recruit circulating monocytes to lungs, and, as they differentiate into macrophages, they upregulate the expression of pro-inflammatory and pro-fibrotic genes, as well as increase antigen presentation to activate T cells and elicit an immune response (Morales-Nebreda et al., 2015). This suggests that the recruitment of macrophages to the lungs during viral infections can disrupt the balance between tissue-resident and recruited macrophages and that monocyte-derived macrophages can be responsible for the cytokine storm and lung tissue damage observed in severe cases of CoV infections. Below, we describe the effects of CoVs in the antiviral response elicited by macrophages.

HCoV-229E can infect macrophages as they express the APN receptor needed for viral entry (Yeager et al., 1992). This virus also can bypass the endosome to enter the target cell by using TMPRSS2 (Shirato et al., 2017). The endosomal pathway in macrophages is critical to identify invading pathogens, as they are the main site for TLRs. Bypassing the endosome allows HCoV-229E to infect macrophages without triggering an antiviral response, thus enhancing its pathogenesis. Human macrophages infected with HCoV-229E undergo cell death due to the lytic release of new viral particles (Collins, 2002), suggesting that HCoV-229E can infect and replicate in macrophages. Additionally, infection of human alveolar macrophages by HCoV-229E showed no production of IFN-β or IL-29, although TNF-α, CXCL10, CCL3, IL-7, and CCL4 were upregulated (Funk et al., 2012). However, the result regarding IFN-β is contradictory to those observed by Cheung et al., where infection of human macrophages by HCoV-229E led to increased IFN-β expression (Cheung et al., 2005). In addition, following infection, macrophages produce and secrete MMP-9, and have increased motility and chemokine-driven migration (Desforges et al., 2007). Besides macrophages, HCoV-229E has cytopathic effects on dendritic cells (DCs) (Mesel-Lemoine et al., 2012), thus negatively impacting the activation of the adaptive immune system and the consequent establishment of long-lasting immunological memory against the virus. This cytopathic effect, together with the ability of the virus to bypass the endosome and suppress interferon signaling, represents a potential mechanism that promotes viral infection and pathogenesis.

Similar to HCoV-229E, SARS-CoV can also infect macrophages, although replication seems to be abortive. The virus can be phagocytosed by macrophages, as it can be detected in phagolysosomes of infected human macrophages (Yilla et al., 2005). When compared to permissive cells, macrophages do not support effective viral replication, as shown by a similar amount of positive and negative RNA in the infected macrophages (Cheung et al., 2005). Although macrophages isolated from patients were found to be enlarged, suggesting macrophage activation (Gu et al., 2005), infected macrophages do not induce an effective antiviral response as they are unable to produce IL-28, IL-29, IFN-α, and IFN-β despite the upregulation of interferon-stimulated genes, and they secrete CXCL10 and CCL2 (Cheung et al., 2005). Interestingly, SARS-CoV proteins were found only in purified monocytes/macrophages that did not produce significant levels of IFN-α (Yilla et al., 2005). Because treatment with interferons decreases SARS-CoV replication (Cinatl et al., 2003; Spiegel et al., 2004), it seems that downregulation of interferon signaling is an advantageous mechanism exploited by SARS-CoV to enhance viral replication and pathogenesis. Besides macrophages, monocyte-derived human DCs also demonstrate incomplete viral replication, low expression of interferons, and moderate upregulation of pro-inflammatory cytokines (Law et al., 2005). In a study with six SARS patients that succumbed to the disease, four post-mortem samples had increased macrophage infiltration in the lungs, and three had multinucleate giant cells of macrophage origin (Nicholls et al., 2003). The induction of M2-like macrophages is also associated with the severity of the disease and enhanced pathogenesis, as observed in a mouse model for SARS (Page et al., 2012). In addition, SARS-CoV-infected epithelial cells can release IL-6 and IL-8, which lead to increased pathogenesis, inhibition of T cell priming, and reduced phagocytic activity of macrophages (Yoshikawa et al., 2009).

Similarly, MERS-CoV can infect monocyte-derived macrophages and DCs, although low replication is observed (Tynell et al., 2016). MERS-CoV can evade the innate immune response by multiple mechanisms. MERS-CoV proteins inhibit type I interferon and NF-κB signaling, and some NSPs can also inhibit dsRNA sensors (Shokri et al., 2019). Other groups demonstrated that the S protein reduces the ability of human macrophages to produce IL-6 and TNF-α, increases IL-10, and induces the expression of negative regulators of TLR signaling via its interaction with dipeptidyl-peptidase 4 (Al-Qahtani et al., 2017). MERS-CoV causes apoptosis in human macrophages, suppresses the proliferation of myeloid progenitor cells, and downregulates antigen presentation, thus inhibiting T cell activation (Shokri et al., 2019). Besides, using a mouse model for MERS, it has been found that early administration of type I interferons protects mice against lethal infection, while delayed treatment increases monocyte and macrophage infiltration in the lungs, increases the production of pro-inflammatory cytokines, and fails to inhibit viral replication (Channappanavar et al., 2016; Channappanavar et al., 2019). This study highlights the importance of the timing in the interferon response to successfully inhibit viral replication and improve outcomes. Furthermore, it suggests that altering the interferon response in M1-like macrophages is advantageous for CoV pathogenicity.

SARS-CoV-2, the causative agent of COVID-19, is the most widely-spread coronavirus to date. Similar to other CoVs, SARS-CoV-2 infection of its target cells depends on ACE2 and TMPRSS2 (Hoffmann et al., 2020), suggesting that SARS-CoV-2 shares some characteristics with SARS-CoV and HCoV-229E. Similar to other CoVs, SARS-CoV-2 also affects macrophage function. Although the information from some reports described below has not been peer-reviewed yet, they provide valuable insight into the understanding of SARS-CoV-2 and macrophage interactions. Interestingly, macrophages exhibit the highest interaction with ACE2+ cells in the lungs, stomach, and liver (Qi et al., 2020). A distinct population of non-classical macrophages is present in the peripheral blood of 28 COVID-19 patients (Zhang D. et al., 2020). This population is composed of monocytes, M1-like, and M2-like macrophages, as demonstrated by flow cytometry analysis where they stained for CD68 and CD80 for M1-like, and CD206 and CD163 for M2-like macrophages, along with other monocyte/macrophage markers. Interestingly, this population had a higher expression of IL-6 and TNF-α and was practically absent in healthy donor controls and samples from other infectious disease patients, e.g., HIV, influenza, or malaria. These results suggest a unique mechanism exploited by CoVs to influence and modulate macrophage function to evade the antiviral immune response.

The importance of macrophages during SARS-CoV-2 infection was reported by another group that demonstrated that ACE2+ macrophages present in the spleen and lymph nodes could be infected by SARS-CoV-2 (Chen Y. et al., 2020). They also found that SARS-CoV-2 enhances the secretion of IL-6 in macrophages and induces apoptosis in lymphocytes through Fas signaling. This suggests that spleen macrophages could be responsible for viral spread, exacerbated inflammation, and subsequent cytokine storm and lymphocytopenia (Park, 2020). IL-6, along with other inflammatory cytokines like TNF-α, IL-1β, and IFN-γ is associated with more severe cases involving macrophage activation syndrome and adult respiratory distress syndrome (McGonagle et al., 2020). In addition, COVID-19 patients with severe respiratory failure have macrophage activation syndrome or dysregulated immune responses, CD4 and natural killer cell cytopenia, and decreased HLA-DR expression in monocytes that can be due to an increase in IL-6 (Giamarellos-Bourboulis et al., 2020). A decrease in T cells has also been observed in severe cases (Qin et al., 2020), along with an increase in neutrophil-lymphocyte ratio and monocyte-lymphocyte ratio (Sun et al., 2020). In two severe cases of COVID-19, lung injuries and cavities were filled with macrophages and neutrophils, where a fibrinous exudate could be detected, along with IL-6, TNF-α, and PD-L1 and a decrease in lymphocytes (Wang et al., 2020). Altogether, these results suggest that increased infiltration of macrophages in the lungs might be responsible for the cytokine storm and pulmonary fibrosis observed in severe cases of COVID-19.

Besides pulmonary fibrosis and failure, other severe complications observed in COVID-19 patients include hypercoagulation or clot formation (Panigada et al., 2020; Spiezia et al., 2020; Terpos et al., 2020). Thrombotic complications occur in 31% of COVID-19 patients that are in the intensive care unit (Klok et al., 2020). Multiple groups have reported that severe COVID-19 patients have increased fibrinogen and D-dimer plasma levels compared to the healthy controls, which might be correlated to the worsening of disease (Panigada et al., 2020; Spiezia et al., 2020; Terpos et al., 2020). Nonsurvivors had higher D-dimer and fibrin degradation products, with 71% of nonsurvivors having intravascular coagulation (Tang et al., 2020). Interestingly, fibrinogen and fibrin can differentially modulate macrophage function. When macrophages are cultured in fibrin gels, they produce anti-inflammatory cytokines like IL-10, while, when exposed to soluble fibrinogen, macrophages release pro-inflammatory cytokines such as TNF-α (Hsieh et al., 2017). In addition, fibrinogen can stimulate the production of TGF-β, activating M2-like macrophages, and increasing collagen production (Vidal et al., 2008). D-dimer can also increase inflammatory responses in macrophages (Zhou et al., 2007). Fibrinogen may engage with TLRs, which can lead to an increase in pro-inflammatory cytokines such as IFN, TNF-α, IL-6, IL-1β, among others, promoting coagulation (Foley and Conway, 2016). These reports indicate the relationship between disrupted macrophage function and the hypercoagulability observed in severely ill COVID-19 patients, which might correlate to the inflammatory profile of these patients.

Although more peer-reviewed research is needed to better understand the implications of SARS-CoV-2 in modulating macrophage function, studies published to date suggest that imbalanced M1-like and M2-like macrophage activity enhances the pathogenesis of CoVs. Therefore, targeting macrophage recruitment and function is a potential approach to treat infections caused by multiple CoVs.

## Therapeutic Targeting of Macrophage-Induced Complications in Coronavirus Disease 2019

There is an urgent need to find therapeutic approaches to halt viral replication, enhance the antiviral immune response, and decrease the aforementioned macrophage-induced complications observed in COVID-19 patients (Figure 2). Current efforts include the investigation of antiviral drugs to decrease viral replication. Among them, ivermectin decreases SARS-CoV-2 replication (Caly et al., 2020), possibly by inhibiting the import of viral proteins (Choudhary and Sharma, 2020). Ivermectin is being investigated in clinical trials for COVID-19 patients, either as a monotherapy or as a combinatorial therapy approach (NCT04381884, NCT04390022, NCT04407507, NCT04392427, NCT04343092). Additionally, chloroquine and hydroxychloroquine are also in clinical trials, alone or in combination with other therapeutic approaches aiming to inhibit viral entry, uncoating, replication, and/or assembly (NCT04331834, NCT04377646). However, substantial efforts are being made to target diverse aspects of macrophage biology, such as interferon response, recruitment, cytokine storm, lung fibrosis, and hypercoagulation in COVID-19 patients. Thus, in this section, we summarize ongoing clinical trials for COVID-19 patients that target macrophage-induced complications in COVID-19 patients (Table 1) and propose novel alternative approaches that could have the same effect (Table 2).

**TABLE 1:** Ongoing clinical trials for COVID-19 patients.

Target	Agent/Drug	^a^ID number Clinicaltrials.gov
Increase interferon response		
IFN-λ	IFN-λ	NCT04343976; NCT04388709; NCT04344600
IFN-β	IFN-β	NCT04350281; NCT04324463
IFN-α	IFN-α	NCT04320238; NCT04379518; NCT04293887
Decrease macrophage recruitment		
GM-CSF and GM-CSF receptor	Lenzilumab	NCT04351152
	Sargramostim	NCT04326920
CCR5	Leronlimab	NCT04343651; NCT04347239
Decrease cytokine storm		
IL-6 and IL-6 receptor	Tocilizumab	NCT04322773
	Sarilumab	NCT04359901; NCT04357808
	Clazakizumab	NCT04348500; NCT04381052; NCT04343989; NCT04381052
	Olokizumab	NCT04380519
IL-1β	Canakinumab	NCT04362813; NCT04348448
TNF	Infliximab	NCT04425538; NCT04344249
	XPro1595	NCT04370236
JAK	Ruxolitinib	NCT04338958; NCT04359290; NCT04348071
	Baricitinib	NCT04340232
TLR4	EB05	NCT04401475
Cytokines	CytoSorb	NCT04344080; NCT04324528; NCT04391920
Inflammatory mediators	Methylprednisolone	NCT04244591; NCT04273321; NCT04329650; NCT04377503; NCT04345445; NCT04341038
	Colchicine	NCT04350320; NCT04375202
	Anakinra	NCT04324021; NCT04443881
Decrease pulmonary fibrosis		
Fibroblast proliferation; collagen deposition; PDGFR, FGFR, VEGFR signaling	Pirfenidone	NCT04282902
	Nintedanib	NCT04338802
Decrease hypercoagulation		
Antithrombin	Enoxaparin	NCT04345848; NCT04377997
	Tinzaparin	NCT04344756
	Heparin	NCT04372589
Factor xa	Bemiparin	NCT04420299
	Rivaroxaban	NCT04416048

IFN, interferon; GM-CSF, granulocyte-macrophage colony-stimulating factor; CCR5, C-C chemokine receptor type 5; IL-6, interleukin 6; IL-1β, interleukin one beta; TNF, tumor necrosis factor; JAK, Janus kinase; PDGFR, platelet derived growth factor receptor; FGFR, fibroblast growth factor receptor; VEGFR, vascular endothelial growth factor receptor.

a Clinical trials only for SARS-CoV-2 or COVID-19. Includes trials that are new, not yet recruiting, recruiting, and/or completed as of August 25, 2020.

**TABLE 2:** Potential alternative approaches to treat macrophage-induced complications in COVID-19 patients.

Target	Drug	Mechanism	Ref
Increase interferon signaling			
TLR7	Imiquimod	Increase in IFN-α, IFN-β, and IFN-γ	(Megyeri et al., 1995; Wang et al., 2005; Huang et al., 2009; O’Brien et al., 2010)
TLR9	CpG oligodeoxy-nucleotides	Increase IFN-α expression and infiltration of CD4 T cells and	(Wang et al., 2005)
TLR3	poly (I:C)	Stimulate RLR pathway through RIG-I to upregulate IFN-β	(Dauletbaev et al., 2015)
dsRNA sensors	DNA methyltransferase inhibitors	Demethylation of endogenous retroviruses triggers dsRNA response, thus increasing IFN-β	(Chiappinelli et al., 2015)
Decrease macrophage recruitment			
CCL2	Bindarit	Decrease CCL2 expression, decrease monocyte/macrophage infiltration	(Baeck et al., 2012; Ge et al., 2012, 2; Mora et al., 2012)
CCL5	Met-RANTES	Reduce inflammatory cell recruitment and cytokine production	(Culley et al., 2006)
CX3CL1/CX3CR1	AZD8797	Decrease inflammation and macrophage activation	(Karlström et al., 2013, 3; Wollberg et al., 2014)
M2-like macrophages	Nexturastat A	Decreases M2-like macrophage recruitment	(Knox et al., 2019; Banik et al., 2020)
Decrease cytokine storm			
Expression of pro-inflammatory cytokines like IL-6, IL-1β, and TNF-α	Trichostatin A	Decreases IL-6 production by modulating mRNA stability	(Grabiec et al., 2011)
		Decrease IL-1β, and TNF-α	(Han and Lee, 2009)
		Decrease systemic inflammation	(Cui et al., 2019)
	CKD-L	Decrease TNF-α and IL-1β	(Oh et al., 2017)
	Tubastatin A	Decrease IL-6 and TNF-α, improves survival, and decreases liver injury	(Li et al., 2015)
	Vorinostat	Reduce cytokine storm induced by administration of anti-CD3 antibodies	(Li et al., 2008)
STAT signaling	Tubastatin A	Decrease STAT3 phosphorylation	(Cheng et al., 2011, 2014)
Decrease pulmonary fibrosis			
Fibroblast viability; expression of fibrogenesis-associated genes	Panobinostat	Decrease gene expression of fibrogenesis-associated genes; induce cell cycle arrest and apoptosis in fibrocytes	(Korfei et al., 2014, 2018)
	Tubastatin A	Decrease TGF-β-induced expression of type I collagen in lung fibroblast	(Saito et al., 2017)
	SAHA	Induce apoptosis in myofibroblasts, decrease pulmonary fibrosis, and increase lung function	(Sanders et al., 2014)

TLR, Toll-like receptor; IFN-α/β/γ, interferon alpha/beta/gamma; RLR, RIG-I-like receptor; dsRNA, double stranded RNA; CCL2/CCL5, C-C motif chemokine ligand 2/5; CX3CL1/CX3CR1, C-X3-C motif chemokine ligand/receptor 1; IL-6, interleukin 6; IL-1β, interleukin 1 beta; TNF-α, tumor necrosis factor alpha; STAT, signal transducer and activator of transcription; TGF-β, transforming growth factor beta; SAHA (suberoylanilide hydroxamic acid.

### Increasing the Interferon Response

Infections with different CoVs have been shown to decrease the interferon response. Therefore, ongoing clinical trials for COVID-19 patients include the administration of IFN-λ (NCT04343976, NCT04388709, NCT04344600), IFN-β (NCT04350281, NCT04324463), and IFN-α (NCT04320238, NCT04379518, NCT04293887) in patients infected with SARS-CoV-2, or in healthcare workers as a preventative measure. Although administering interferons might be a practical approach to induce an antiviral response, either when administered as a therapy or as a preventative measure, there are alternative approaches to increase the interferon response. Among them, TLR agonists, some of which are approved by the U.S. Food and Drug Administration, could be used for this purpose. For example, the TLR7 agonist imiquimod, a synthetic analog of single-stranded RNA, increases the expression of IFN-α, IFN-β, and IFN-γ, and enhances T cell effector function (Megyeri et al., 1995; Huang et al., 2009; O’Brien et al., 2010). Also, imiquimod and the TLR9 agonist CpG oligodeoxynucleotide can both increase IFN-α expression and infiltration of CD4 T cells and DCs (Wang et al., 2005). In addition, poly (I:C) is a TLR3 agonist that induces antiviral immune responses for respiratory viruses while stimulating the RLR pathway through RIG-I to upregulate IFN-β (Christopher and Wong, 2011; Dauletbaev et al., 2015). Other approaches to increase interferon signaling by triggering dsRNA sensors include inhibiting DNA methyltransferases (Chiappinelli et al., 2015) or targeting RLRs (Yong and Luo, 2018). Other approaches stimulating RLRs are reviewed in the manuscript by Yong HY and Luo D (Yong and Luo, 2018). Also, histone deacetylase 6 (HDAC6) could be a potential target as its genetic knockdown or inhibition with Tubastatin A enhances the release of IFN-γ in isolated T cells and peritoneal macrophages (Cheng et al., 2014).

### Decreasing Macrophage Recruitment

This can be achieved by administering anti-CSF1-R or anti-GM-CSF antibodies to prevent the differentiation of monocytes into macrophages. For example, lenzilumab, an anti-GM-CSF antibody, is in clinical trials for COVID-19 patients (NCT04351152). In addition, sargramostim, a human-made form of GM-CSF that binds to the GM-CSF receptor, is under investigation for the same purpose (NCT04326920). Another alternative is the use of anti-CCR5 to prevent the recruitment of macrophages to the infection site. Leronlimab, an anti-CCR5 antibody, is currently in clinical trials for the treatment of COVID-19 patients (NCT04343651; NCT04347239).

Although the antibodies being tested in clinical trials are great options, there are alternative approaches to decrease macrophage recruitment, thus reducing the macrophage-derived complications observed in COVID-19 patients. A potential approach includes the inhibition of chemokines or their receptors (e.g., CCL2, CCL5, CX3CL1, and chemerin, among others). CCL2 can recruit macrophages and neutrophils, increasing lung inflammation after infection (Balamayooran et al., 2011; van Zoelen et al., 2011). Bindarit is a small molecule that modulates the NF-κB pathway and decreases CCL2 expression (Ge et al., 2012; Mora et al., 2012). Pharmacological inhibition of CCL2 decreases monocyte/macrophage infiltration in the liver (Baeck et al., 2012). Although tested in a liver injury model, these principles could be applied to COVID-19 to decrease monocyte/macrophage recruitment into the lung, thus decreasing inflammation and pulmonary fibrosis. In addition to CCL2, CCL5 (also called RANTES), is another well-characterized chemoattractant for macrophages (Lee et al., 2017). Previous research has shown that Met-RANTES, a potent chemokine receptor blocker (Proudfoot et al., 1996), reduces inflammatory cell recruitment and cytokine production (Culley et al., 2006). CX3CL1 is another macrophage chemoattractant that is expressed by fibroblasts and macrophages in the lungs, and it is upregulated in pulmonary fibrosis (Hasegawa et al., 2005; Zhang and Patel, 2010, 3). AZD8797, an inhibitor for the receptor for CX3CL1, decreases inflammation and macrophage activation (Karlström et al., 2013; Wollberg et al., 2014). In addition, chemerin is a chemoattractant expressed in the lungs that recruits DCs and macrophages to the site of inflammation (Wittamer et al., 2003). Besides these chemokines, CD44 also regulates macrophage recruitment to the lungs in an LPS-induced disease model (Hollingsworth et al., 2007). Inhibition of the CD44 receptor could be a potential mechanism to prevent macrophage recruitment to the lungs in the context of SARS-CoV-2 infection. CD44 inhibitors are being developed and characterized (Harada et al., 2006).

### Decreasing Cytokine Storm

Neutralizing IL-6 or its receptor by using anti-IL-6 or anti-IL-6R antibodies, respectively, is being currently investigated to help severe cases of COVID-19 that suffer from complications derived from cytokine storm (Ascierto et al., 2020; Buonaguro et al., 2020; Luo et al., 2020; Michot et al., 2020; Xu X. et al., 2020; Zhao, 2020; Zhang X. et al., 2020). Anti-IL-6R antibodies such as tocilizumab (NCT04322773) or sarilumab (NCT04359901; NCT04357808), and the anti-IL-6 antibodies clazakizumab (NCT04348500; NCT04381052; NCT04343989; NCT04381052) and olokizumab (NCT04380519) are currently in clinical trials for COVID-19 patients. To date, severe cases of COVID-19 treated with tocilizumab have shown encouraging results. In two reports with 15 and 20 patients each, COVID-19 patients treated with anti-IL-6 antibodies had C-reactive protein and lymphocyte levels returning to normal after treatment (Luo et al., 2020; Xu X. et al., 2020). Full recovery from COVID-19 was also reported in a renal cell carcinoma patient treated with antiviral therapy and tocilizumab (Michot et al., 2020), thus demonstrating the efficacy of this approach to reduce the life-threatening effects of the cytokine storm. Besides IL-6, IL-1β and TNF-α are also responsible for the cytokine storm. Anti-IL-1β antibodies such as canakinumab are being tested in clinical trials for COVID-19 patients (NCT04362813; NCT04348448). A retrospective analysis of 10 patients treated with canakinumab reported the efficacy this therapy to decrease C-reactive protein and provide full recovery from the disease (Ucciferri et al., 2020). Also, targeting TNF-α with neutralizing antibodies is a potential therapeutic approach, as their safety has been proven in a variety of diseases (Feldmann et al., 2020). TNF-α neutralizing antibodies reduce the recruitment of inflammatory cells such as neutrophils (Jones et al., 2005) as well as cytokine production in the lungs without affecting clearance of the virus (Hussell et al., 2001). Although clinical trials using anti-TNF-α antibodies for COVID-19 patients are limited to the monoclonal antibody infliximab (NCT04425538; NCT04344249), the evaluation of XPro1595, which prevents TNFs from binding to their receptors by forming heterotrimers with soluble TNF (Steed et al., 2003), is under current evaluation for COVID-19 patients (NCT04370236). In addition, CytoSorb, a safe and well-tolerated (Mehta Y. et al., 2020) cytokine adsorber, is under investigation in clinical trials to reduce cytokine storm and inflammatory mediators in the blood of severely ill COVID-19 patients (NCT04344080; NCT04324528; NCT04391920).

Besides neutralizing cytokines, the signaling pathways triggered by the engagement of these cytokines with their receptors can be inhibited. It is well known that most cytokines signal through the Jak/STAT pathway, thus Jak and STAT inhibitors are potential targets to suppress the cellular response elicited by these cytokines. For example, the Jak1/Jak2 inhibitor ruxolitinib is currently under investigation in clinical trials in severe COVID-19 patients (NCT04338958; NCT04359290; NCT04348071). Baricitinib, another Jak1/Jak2 inhibitor, is also under investigation for COVID-19 patients (NCT04340232). A different approach that is also under investigation is the inhibition of TLR4 signaling. At the time of this review, one clinical trial is already using a TLR4 inhibitor (EB05; NCT04401475) as an approach to reduce the cytokine storm and subsequent lung damage in COVID-19 patients.

Furthermore, immunosuppressive drugs are being investigated in clinical trials for COVID-19 patients. For example, methylprednisolone is a corticosteroid that reduces the expression of pro-inflammatory cytokines, decreases the cytopathic effects of NO (nitric oxide) and TNF-α, inhibits T cell activation, and decreases the extravasation of immune cells (Sloka and Stefanelli, 2005). For these purposes, methylprednisolone is being investigated alone (NCT04244591, NCT04273321), or in combination with siltuximab (NCT04329650), tocilizumab (NCT04377503, NCT04345445), or tacrolimus (NCT04341038). Other anti-inflammatory drugs include colchicine (NCT04350320; NCT04375202) and anakinra (NCT04324021; NCT04443881).

Besides these strategies, other alternative approaches should be considered. For instance, epigenetic modifiers like HDAC inhibitors can have immunomodulatory effects, influencing the cellular responses triggered by cytokine signaling. Pharmacological inhibition and genetic knockdown of HDAC6 can decrease STAT3 phosphorylation, leading to the downregulation of some STAT3 target genes (Cheng et al., 2011; Cheng et al., 2014; Lienlaf et al., 2016). Additionally, HDAC inhibitors can decrease the expression of pro-inflammatory cytokines. For example, Trichostatin A suppresses IL-6 production in macrophages by modulating the stability of its messenger RNA without affecting NF-κB activation (Grabiec et al., 2011). Trichostatin A also decreases the production of TNF-α and IL-1β while increasing the production of the immunosuppressive cytokine IL-10 in bone marrow-derived macrophages (Han and Lee, 2009), and reducing systemic inflammation (Cui et al., 2019). Other inhibitors, like the HDAC6 inhibitors CKD-L and Tubastatin A showed similar results. To illustrate, CKD-L decreases the expression of TNF-α and IL-1β and increases IL-10 in a collagen-induced arthritis model (Oh et al., 2017), and Tubastatin A also modulates the expression of those cytokines, while increasing survival and protecting mice from acute liver injury in a lethal septic model (Li et al., 2015). Although the studies with Tubastatin A may help explain the role of HDAC6 in regulating cytokine expression, this inhibitor has reduced bioavailability and cannot be orally administered to patients (Cosenza and Pozzi, 2018). Importantly, Vorinostat, an HDAC inhibitor approved by the Food and Drug Administration, decreases cytokine storm induced by anti-CD3 antibodies (Li et al., 2008). Overall, these reports suggest that HDAC inhibitors could be used to decrease the expression of the pro-inflammatory cytokines that lead to the cytokine storm in patients without potentially affecting other target genes of NF-κB, such as interferons. More importantly, HDAC inhibitors could be used as adjuvants for the antibody-based therapies that are in clinical trials for COVID-19 patients. While the HDAC inhibitors would decrease the expression of IL-6, TNF-α, and IL-1β in macrophages, the antibodies would mitigate the systemic effects of these cytokines or decrease macrophage recruitment, depending on antibody specificity.

### Decreasing Pulmonary Fibrosis

Because lung damage and consequent fibrosis occur in COVID-19 patients due to lung injury and/or pneumonia caused by SARS-CoV-2 (Xu Y.-H. et al., 2020), decreasing lung fibrosis could be beneficial to decrease the long-lasting effects that the infection may have in patients. Besides lung transplantation (Chen et al., 2020), other approaches can be taken to mitigate fibrosis. Some pharmacological approaches include pirfenidone and nintedanib, both of which are being investigated in clinical trials for COVID-19 patients (NCT04282902, and NCT04338802, respectively). These compounds impair fibroblast proliferation and migration, myofibroblast differentiation, collagen fibril assembly, and signaling through PDGFR, FGFR, and VEGFR (Wollin et al., 2015; Knüppel et al., 2017; Kurita et al., 2017).

However, other approaches must be taken into consideration to prevent or decrease lung fibrosis in COVID-19 patients. While the approaches being investigated in clinical trials target fibroblasts, M2-like macrophages play crucial roles in the fibrotic process. Panobinostat, a pan-HDAC inhibitor, decreases the expression of fibrogenesis-associated genes in a pulmonary fibrotic model (Korfei et al., 2014). Panobinostat induces cell cycle arrest and apoptosis in fibrocytes and decreases lung fibrosis more efficiently than pirfenidone (Korfei et al., 2018). Treatment with SAHA (suberoylanilide hydroxamic acid) induces apoptosis in myofibroblasts, decreases lung fibrosis, and improves lung function in a pulmonary fibrosis mouse model (Sanders et al., 2014). More information on the use of HDAC inhibitors as antifibrotic drugs can be found in the review by Lyu X, et al. (Lyu et al., 2019). Isotype-selective HDAC6 inhibitors decrease TGF-β-induced expression of type I collagen in lung fibroblasts (Saito et al., 2017). Additionally, HDAC6 inhibition significantly decreases M2-like macrophages (Knox et al., 2019; Banik et al., 2020), thus suggesting that HDAC6 inhibitors could be used to decrease M2-like macrophage-driven fibroblast activation. Besides, HDAC6 plays an essential role in viral entry (Banerjee et al., 2014) and innate immunity (Moreno-Gonzalo et al., 2018). Although epigenetic modifiers are not in clinical trials for COVID-19 patients at the moment of writing this review, they represent an attractive approach as they can regulate multiple cellular processes. For example, they can decrease pulmonary fibrosis, M2-like macrophage polarization, fibrocyte viability, TGF-β signaling, and expression of fibrogenesis-associated genes, among others.

### Decreasing Hypercoagulation

Members of the coagulation cascade can influence the macrophage phenotype. Hence, it is crucial to decrease hypercoagulation in severe COVID-19 cases to prevent the damage associated with this condition and to modulate macrophage function indirectly. Therapies targeting hypercoagulation or blood clotting are being investigated in clinical trials as a therapeutic approach and as a preventive measure. Some anticoagulants antithrombotic drugs being investigated include enoxaparin (NCT04345848, NCT04377997), tinzaparin (NCT04344756), heparin (NCT04372589), bemiparin (NCT04420299), and rivaroxaban (NCT04416048). In addition, other clinical trials are investigating the combination of multiple anticoagulants for COVID-19 (NCT04394377). Overall, these anticoagulant drugs activate negative regulators of the coagulation cascade or directly inhibit the activity of different members of the coagulation cascade. To illustrate, enoxaparin, tinzaparin, and heparin bind to antithrombin, which then becomes activated and, in turn, inactivates pro-coagulation factors such as Factor Xa and thrombin. In contrast, bemiparin and rivaroxaban directly inhibit Factor Xa.

## Final Remarks

Although data regarding the immune response in COVID-19 patients is becoming available at the time of writing this review and some reports cited here are pre-prints, it is clear that CoVs negatively affect the antiviral immune response elicited by macrophages, as this can be observed in data describing other CoVs like HCoV-229E, SARS-CoV, and MERS-CoV. In summary, CoVs reduce the interferon response in M1-like macrophages while increasing the production and secretion of pro-inflammatory cytokines such as IL-6, TNF-α, and IL-1β that cause the cytokine storm in patients. Due to the effects of these cytokines in other immune cells, along with an ineffective antiviral immune response and decrease in antigen presentation, the adaptive immune response remains poorly effective. Besides, the overproduction of cytokine storm mediators creates positive feedback loops that promote M1-like macrophage function and disrupts tissue integrity, leading to tissue damage. Excessive IL-6 can cause M1-like macrophages to switch their phenotype to M2-like. M2-like macrophages initiate the resolution of inflammation and attempt to heal the damaged tissue, partially by activating or transitioning to myofibroblasts, which secrete collagen and MMPs. Due to the imbalance in macrophage function and fibroblast activity, pulmonary fibrosis and scarring occur in patients. If the patient survives the cytokine storm, this fibrotic and scarring process would likely have long-life complications.

Due to the role of macrophages in the pathogenesis of CoVs and in cytokine storms, fibrosis, and hypercoagulation, numerous clinical trials are trying to identify effective therapies that can mitigate the aforementioned macrophage-induced complications. These approaches include stimulating the antiviral immune response, preventing macrophage recruitment, and decreasing cytokine storm, pulmonary fibrosis, and hypercoagulation. Although some of the therapies that are currently being investigated in clinical trials might be effective against SARS-CoV-2 infection, more research is needed to develop other therapeutic approaches or combinatorial therapies that can further mitigate these effects while eliciting an effective immune response and memory. For these purposes, alternative approaches like the ones summarized in Table 2 should be taken into consideration. For example, treatment with epigenetic modifiers could decrease the expression of pro-inflammatory cytokines that lead to the cytokine storm, while also targeting M2-like macrophage polarization and activity to decrease the signaling involved in the fibrotic process. Furthermore, thanks to their immunomodulatory effects, epigenetic modifiers could be potentially used in combination with other therapies that are currently being investigated in clinical trials.

Although clinical trials are ongoing with the therapies described here, some early results (i.e., anti-IL-6 antibodies) are encouraging as they seem to decrease patient mortality and provide full recovery in some patients. While researchers around the globe are seeking to develop and test potential vaccines against COVID-19, in the meantime, efforts should be devoted to identifying novel therapies that either alone or in combination could halt disease progression, enhance the antiviral immune response, modulate macrophage activity, and improve the outcome of COVID-19 patients.

## Author Contributions

MH: writing, coordinating, making tables and figures, editing, inserting references; ES: coordinating, reviewing, editing, proofreading; AV: coordinating, writing, reviewing, editing, proofreading.

## Conflict of Interest

The authors declare that the research was conducted in the absence of any commercial or financial relationships that could be construed as a potential conflict of interest.
